# From Launch to Legacy: Charting the Path of the Intergovernmental Science-Policy Panel on Chemicals, Waste, and Pollution

**DOI:** 10.1021/acs.est.5c18231

**Published:** 2026-05-27

**Authors:** Viola Pavoncello, Simone Marzeddu, Lucilla Baldassarri, Daniele Bianconi, Valerio Paolini, Silvia Mosca, Serena Santoro

**Affiliations:** † Institute of Atmospheric Pollution Research (IIA), National Research Council of Italy (CNR), Montelibretti, Via Salaria (km 29.300), 00010 Rome, Italy; ‡ Hazardous Substances Section, Directorate for Environmental Education, Training and Technical Coordination Activities (DG-TEC), Italian Institute for Environmental Protection and Research (ISPRA), Via Vitaliano Brancati 48, 00144 Rome, Italy; § National Centre for Chemicals, Cosmetics and Consumer Protection (CNSC), Italian National Institute of Health (ISS), Via Giano della Bella 34, 00161 Rome, Italy

**Keywords:** science-policy interface, ISP-CWP, global chemicals, waste agenda, pollution prevention, multilateral environmental agreements, horizon-scanning, environmental governance

## Abstract

The establishment of the Intergovernmental Science-Policy Panel on Chemicals, Waste and Pollution (ISP-CWP) marks the first creation of a dedicated intergovernmental science-policy body addressing the sound management of chemicals and waste and the prevention of pollution. Emerging within a governance landscape shaped by a variety of longstanding international frameworks, the Panel seeks to strengthen the interface between scientific knowledge and decision-making in a domain characterized by diverse institutional actors, uneven capacities, and economic implications. This perspective analyzes the institutional conditions that will determine whether the ISP-CWP can translate its formal mandate into effective governance. We examine how its mandate, governance architecture, and core functions—including horizon scanning, solution-oriented assessments, knowledge inclusivity, and integrated capacity-building—may shape the Panel’s credibility, legitimacy, and policy relevance in practice. While the Panel’s design reflects lessons learned from earlier science-policy bodies, it also embeds structural tensions: between scientific independence and political oversight, timeliness and methodological rigor, inclusivity and operational feasibility. The ISP-CWP’s long-term influence will depend less on its formal mandate than on how effectively it manages these trade-offs in practice. As such, the Panel represents a live experiment for the evolution of science-policy interfaces in politically sensitive and economically complex environmental domains.

## Graphical abstract

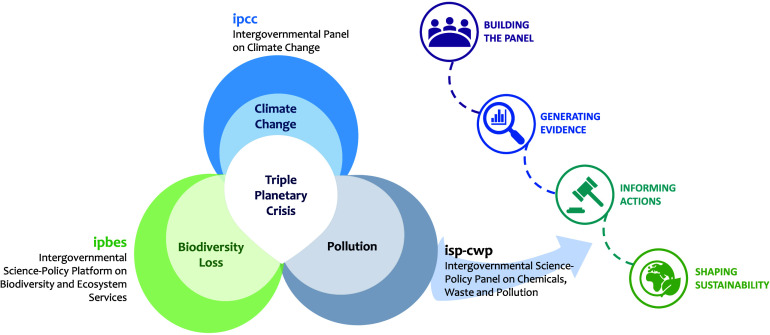

## Introduction

Our planet is facing an interconnected “triple crisis”: climate change, biodiversity loss, and pollution.^1^ While the first two challenges have long been at the center of global attention, with dedicated science-policy bodies such as the Intergovernmental Panel on Climate Change (IPCC) and the Intergovernmental Science-Policy Platform on Biodiversity and Ecosystem Services (IPBES),^2^ the third component has suffered from a fragmented and less coordinated approach.^3,4^ Indeed, pollution prevention, combined with the sustainable management of chemicals and waste, has become a critical priority for protecting human health and the environment,^5^ and the Panel arrives at a critical juncture. Current data indicate a rapidly growing production of industrial chemicals, with levels projected to double by 2030 compared to 2017.^6^ More than 350,000 chemicals are registered for use globally, with many poorly characterized in terms of risks to human and ecological health.^7,8^ A similar trend is also true for wastes.^6^ Waste streams are intensifying, from plastics and e-waste to pharmaceuticals and pesticides. Data reported that global municipal solid waste is expected to increase dramatically from 2.1 billion tonnes in 2023 to 3.8 billion tonnes by 2050. In 2020, the direct global cost of waste management was estimated at USD 252 billion; including the hidden costs of pollution, health impacts, and climate change, the total rises to USD 361 billion.^9^

Pollution contributes to non-communicable diseases and undermines ecosystem integrity,^10,11^ while imposing substantial economic costs through lost productivity, healthcare burdens, and degraded natural capital, which further exacerbate social and economic inequalities (Figure 1).^4^ These observations underscore the critical need for a dedicated science-policy interface to chemical, waste and pollution prevention, that not only leverages and generates knowledge but actively translates it into governance mechanisms, bridging the gap between research and decision-making. By situating the ISP-CWP in this context, we emphasize its potential to address previously fragmented approaches and provide a coherent framework for global chemical, waste and pollution management.

**Figure 1:**
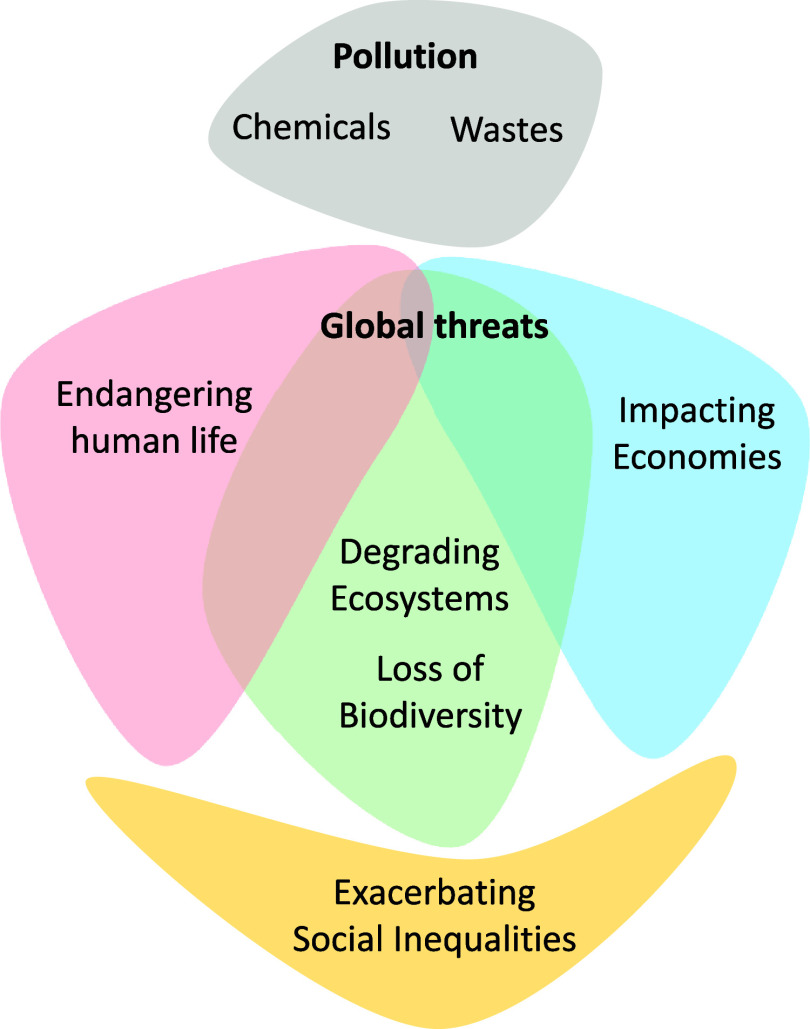
The triple threat of pollution and its social consequences. Pollution from chemicals and waste exerts interconnected pressures on human health, ecosystem biodiversity, and economic systems, each of which can independently and collectively exacerbate social and economic inequalities, particularly in low- and middle-income countries (LMICs). The figure illustrates these three primary domains as overlapping elements, with the bottom ends converging on social inequalities to highlight their cumulative impact. The ISP-CWP has the potential to be a central integrative hub, synthesizing scientific knowledge across domains, performing horizon scanning, and producing solution-oriented assessments to inform policy. By linking scientific evidence to governance mechanisms and other Multilateral Environmental Agreement (MEAs), the Panel has the potential to mitigate the cascading effects of pollution on human, ecological, and economic systems, while promoting more equitable outcomes globally.

In this context, the United Nations Environment Assembly (UNEA), through resolution 5/8 adopted on 2 March 2022, recognized the need to establish a dedicated science-policy panel on the sound management of chemicals and waste and on pollution prevention.^12^ Following this mandate, on 20 June 2025 in Punta del Este, Uruguay, governments formally launched the Intergovernmental Science-Policy Panel on Chemicals, Waste and Pollution (ISP-CWP, hereafter “the Panel”).^13^ The international community has laid the foundations for an authoritative intergovernmental body to strengthen the science-policy interface on chemicals, waste, and pollution prevention, addressing a component of the broader environmental challenges that complement climate change and biodiversity loss. Unlike climate and biodiversity, where centralized assessment bodies have gradually consolidated scientific authority, chemicals and waste governance has evolved through a set of Multilateral Environmental Agreements (MEAs), voluntary frameworks, and national regulatory systems with uneven regional capacities and influence. This fragmentation has limited anticipatory governance, slowed collective responses to emerging risks, and amplified asymmetries between countries with advanced regulatory infrastructures and those lacking scientific and technical resources.^3^

While previous contributions have emphasized the importance of establishing a dedicated science-policy panel on chemicals and pollution,^14^ less attention has been devoted to examining how its emerging architecture may shape the Panel’s credibility, legitimacy, and policy relevance. This Perspective therefore moves beyond advocacy-oriented discussions and provides an analytical assessment of the ISP-CWP’s developing governance structure. Drawing on recent scholarship on science-policy interfaces,^1,15^ we examine what the ISP-CWP is mandated to do, and how its institutional architecture attempts to reconcile ambition with structural constraints. We analyze the Panel’s design as a governance innovation shaped by trade-offs between scientific independence and political oversight, inclusivity and operational feasibility, solution-orientation and nonprescriptive advisory functions. In doing so, we position the ISP-CWP as a critical test case for the evolution of science-policy interfaces in politically sensitive and economically complex policy domains. By situating the ISP-CWP within the broader landscape of intergovernmental bodies, we assess whether it primarily fills a long-standing institutional gap or signals a systemic shift toward a more integrated and solution-oriented model of global environmental governance.

## From Establishment to Institutionalization of the ISP-CWP

The global pollution crisis, disproportionally affecting low- and middle-income countries (LMICs), highlighted the need for a dedicated science-policy interface to strengthen evidence-based chemicals and waste governance. In response, UNEA Resolution 5/8 (2022) mandated the creation of an Intergovernmental Science-Policy Panel on chemicals, waste, and pollution prevention.^16^ Between 2022 and 2025, UNEP convened a series of Open-Ended Working Groups (OEWGs) to define the Panel’s structure, governance principles, and operational procedures (Figure 2). Across five OEWG meetings, negotiations revealed underlying tensions that continue to shape the Panel’s architecture.^7,17^ Debates over the composition, structure and authority of the Interdisciplinary Expert Committee (IEC) reflected broader concerns about balancing scientific autonomy with intergovernmental oversight.^17^ Discussions on observer participation and stakeholder engagement exposed differing views on how open the Panel should be to non-state actors, particularly given the economic stakes associated with chemicals production and waste management. Similarly, protracted negotiations on gender parity, Indigenous knowledge recognition, and regional representation underscored that inclusivity is not merely a normative aspiration but a politically negotiated design feature.^18^

**Figure 2:**
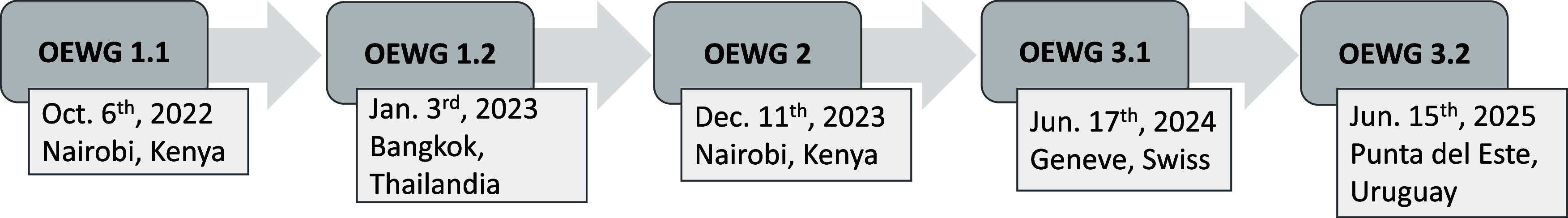
Timeline of the Open-Ended Working Group (OEWG) following UNEA Resolution 5/8 (2022). The diagram presents the sequence of OEWG meetings convened by UNEP from 2022 to 2025 to prepare the foundational documents for the establishment of the ISP-CWP. Over five meetings across three sessions, the OEWG developed proposals on the Panel’s structure, functions, operational principles, and financing. The timeline highlights the Panel’s multiyear negotiation and consensus-building process that culminated in its formal establishment at the Intergovernmental Meeting on June 20^th^, 2025.

The negotiations illustrate that the ISP-CWP’s mandate is the product of compromise: advisory rather than regulatory, solution-oriented yet formally non-prescriptive, and globally scoped yet dependent on voluntary state support.

The ISP-CWP was formally established at the Intergovernmental Meeting on 20 June 2025 by 98 governments.^19,20^ Progress toward the Panel was formally acknowledged by the UNEA at its seventh session (Nairobi, 8–12 December 2025), through decision UNEP/EA.7/6 on the implementation of resolution 5/8.^21^ Therefore, the Panel’s authority will rest less on formal mandate alone than on how effectively it institutionalizes credibility and legitimacy in practice. The first Plenary (2–6 February 2026, Geneva) initiated negotiations on procedural rules, financing mechanisms, and expert selection modalities, marking the shift from formal establishment to operational governance.^22^

## Innovative Features and Approach

Unlike existing interface bodies under global intergovernmental organizations and MEAs that often focus on specific chemicals (e.g., the Stockholm Convention on Persistent Organic Pollutants), regions (e.g., the Bamako Convention for Africa), or lifecycle stages (e.g., the Basel Convention on the transboundary movement and disposal of hazardous waste), the ISP-CWP adopts an integrated, inter- and multidisciplinary approach.^14^ The Panel may address chemicals, waste, and pollution prevention in a cross-cutting manner, producing solution-oriented outputs that support actionable interventions in practice, such as environmental monitoring, waste management, remediation strategies, chemical risk assessment, and human biomonitoring.^17,23^ These interventions may include the development of technical guidance, harmonized analytical methods, and updated chemical risk assessment methodologies. Foundational contributions from the independent International Panel on Chemical Pollution informed the Panel’s establishment through white papers on policy instruments, regulatory innovations, and technological options.^1,15^ The Panel ambition is now codified in the official negotiating text; Figure 3 provides a schematic overview of the five core, interconnected functions that define the ISP-CWP’s operational architecture.

**Figure 3:**
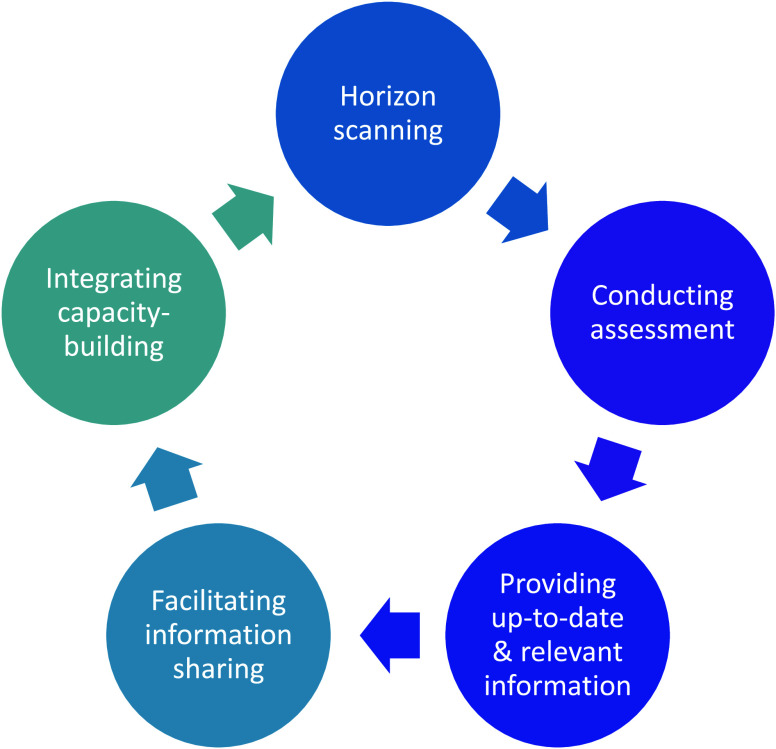
Core functions of the ISP-CWP. Cycle diagram illustrating the five core, interconnected functions of the ISP-CWP: horizon scanning, conducting assessments, providing up-to-date and relevant information, facilitating information sharing, and integrating capacity-building. The circular structure emphasizes their iterative and reinforcing nature, with horizon scanning—new relative to IPCC and IPBES—feeding emerging issues into assessments, while information flows and capacity-building strengthen effective, inclusive science-policy engagement. Together, these functions position the ISP-CWP as a dynamic and solutions-oriented global science-policy mechanism. (Informed by materials presented by UNEP during the “Road to SPC–CWP P1” webinar).^29^

The Panel’s first core, innovative function “*undertaking horizon scanning to identify issues of relevance to policymakers* [...]” (CRP.5, Annex I, para 1­(a))^24^ tasks the ISP-CWP with identifying emerging issues of relevance and proposing evidence-based response options. Institutionalizing horizon scanning formalizes the Panel capacity to anticipate emerging threats and identify new opportunities for sound chemicals and waste management, thereby strengthening the proactive dimension of global governance.^25,26^ Well-defined procedures for systematically reviewing diverse knowledge domains, together with clearly assigned roles and responsibilities and institutional capacity will enable the effective implementation of horizon scanning across the Panel’s wide scope.^26^ However, once identified, prioritizing emerging issues inevitably involves value judgments regarding risk perception, economic impact, and regulatory feasibility. The extent to which horizon scanning outputs will translate into agenda-setting within existing MEAs or national frameworks remains uncertain.

According to the draft decision, the Panel’s second and third core functions include “conducting *assessments of current issues and identifying potential evidence-based options to address (them)* [...]”, and “*providing up-to-date and relevant information, identifying key gaps in scientific research* [...]” (CRP.5, Annex I, para 1­(b,c)).^24^ These formalize a shift toward global assessments that not only diagnose problems but also present solution pathways.^27^ The Panel aims to support strategies that consider the full lifecycle of chemicals and waste, from production to disposal and potential reuse, in line with circular economy principles.^12^

Information sharing and knowledge inclusivity are central: the Panel aims to systemically integrate sectoral, scientific, technical, Indigenous voices and local expertise, with the aim to reflect regional and local contexts (CRP.5, Annex I, para 1­(d), and para 2­(e,f)).^24^ This principle is enshrined in the draft decision text, which specifies that deliverables must consider not only peer-reviewed literature but also “*Indigenous Peoples and local knowledge holders can serve as primary sources of data and information that may be of direct relevance to deliverables* [...]” (CRP.6, Annex III, para. 37–38).^28^ While the formal recognition of Indigenous, local and practice-based knowledge represents an important step forward, operationalizing meaningful participation will require procedural efforts through the establishment of formal participation pathways, engagement frameworks and community monitoring, adequate resources to enable their effective participation, and mechanisms to prevent symbolic involvement. Establishing clear mechanisms and criteria for systematically screening across diverse knowledge domains will be also essential for operationalizing successfully horizon scanning within the Panel’s broad thematic remit.^26^ Without such safeguards, inclusivity risks remaining declarative rather than really integrated into the Panel’s work.

Ensuring scientific credibility, legitimacy, and policy relevance is a cornerstone. Proposed conflict-of-interest (COI) policies require disclosure from all experts and staff, with independent review to safeguard impartiality.^15^

Embedding capacity-building, that encompasses technical assistance, institutional strengthening, digital transformation and technology transfer, within the Panel’s core mandate expands its role beyond traditional assessment function.^15^ The draft decision text states “*integrat­[*e*] capacity-building into all functions and the work of the Panel to strengthen the science-policy interface* [...] *guided by the capacity-building priorities identified by governments and other stakeholders, particularly in developing countries*” (CRP.5, Annex I, para 1­(e)).^24^ Yet this integration may stretch institutional capacity and create dependencies on sustained and predictable financing, raising questions about long-term feasibility.

Taken together, these features position the ISP-CWP as a solutions-oriented, adaptive mechanism. By operating through a continuous cycle of knowledge generation, assessment, sharing, and capacity building, the Panel is designed to respond dynamically to emerging challenges while supporting coherent, evidence-based action (Figure 3). However, this adaptive ambition will depend on procedural choices that remain politically contested, including the frequency of Plenary sessions.^22^ Infrequent intergovernmental meetings could constrain responsiveness to rapidly evolving chemical risks, potentially narrowing the space for timely science-policy interaction.

## Opportunities Ahead

With its integrated, solution-oriented mandate, formally defined to include identifying evidence-based options and disseminating findings, the Panel is exceptionally positioned to evaluate policy instruments, regulatory innovations, and technological options across diverse contexts.^24^ The ISP-CWP has the potential to function as a connective hub across otherwise siloed agreements, also by aggregating and leveraging existing knowledge to inform national and regional policies.^26^ It may have the capacity to inform ongoing negotiations—plastics governance, MEAs and voluntary frameworks such as the Global Framework on Chemicals.^30^ Nevertheless, without clear pathways for integrating its findings into policy processes, even high-quality assessments may have limited influence on international, regional and national governance processes. Importantly, the ISP-CWP appears well-positioned to address several key gaps previously identified in global chemicals and waste governance, including the lack of comprehensive coverage, limited horizon scanning, insufficient bidirectional communication, and underrepresentation of the scientific community.^14,15^ Solution-oriented assessments could support harmonization of human biomonitoring methodologies or inform best practices in hazardous waste remediation in regions lacking technical infrastructure. Integrated horizon scanning outputs could identify contaminants, such as new PFAS substitutes or microplastic additives, allowing governments to coordinate precautionary responses before risk become widespread.

Structured stakeholder engagement—ranging from governments to scientific communities and civil society—can facilitate iterative exchanges in which scientific assessments inform policy decisions while policy needs and societal concerns help shape research priorities and knowledge production.^31,32^ However, it may also introduce procedural complexity and slower decision-making, particularly when divergent interests are at stake.

Finally, the involvement of scientific experts—from multidisciplines and multisectors^17^—through the IEC, alongside proposed COI safeguards, addresses concerns about credibility and inclusion of the scientific community. While these design elements are promising, their effectiveness will hinge on how tensions between scientific independence and political pressures are managed, and whether stakeholder engagement can be truly meaningful across regions with differing capacities. Whether the ISP-CWP marks a paradigm shift will depend on its ability to translate institutional design into sustained influence across fragmented governance fora.

## Immediate Challenges

The Panel’s ambitions will be tested early by structural constraints embedded in its design. Sustainable financing is perhaps the most immediate concern. Reliance on voluntary contributions risks uneven participation, delayed assessment cycles, and limited engagement from LMICs, potentially undermining both equity and global relevance.

Scientific independence presents a second critical challenge. Chemicals and waste governance operates within sectors by significant industrial and economic interests. As documented in prior analyses of COI in environmental assessment, influence is often structural rather than episodic.^33^ Robust disclosure requirements, transparent expert selection procedures, and clear recusal mechanisms will therefore be essential not merely as formal safeguards, but as ongoing institutional practices.^17,33^

A further tension concerns the balance between timeliness and rigor. Governments require responsive guidance on emerging chemical risks, yet the Panel’s legitimacy will depend on meticulous peer review and methodological transparency. Compressing assessment timelines may enhance policy relevance but could also expose the Panel to criticism if perceived as insufficiently robust.

Finally, global asymmetries remain central. Chemical’s production, waste trade, regulatory capacity, and exposure burdens are unevenly distributed across regions. Ensuring meaningful participation from countries with limited scientific infrastructure will require more than formal representation, it will demand sustained investment in capacity-building and inclusive procedural design.

If the ISP-CWP aims to exercise durable authority, its effectiveness should also be subject to systematic evaluation. Possible indicators include the extent to which its assessments inform MEAs, the accessibility and usability of its outputs for policymakers, and the degree of equitable participation across regions and knowledge systems. However, measuring such impact presents methodological challenges. Policy uptake is often indirect, diffuse, and subject to long time lags, making it difficult to attribute results. Any evaluative framework will therefore need to balance measurable outputs with qualitative assessments of the Panel’s scientific and institutional influence. Taken together, these challenges underscore that the ISP-CWP’s authority will not be self-executing. It will need to be constructed through practice, transparency, and demonstrable fairness in its early years.

## Comparative Lessons

The new Panel does not start from scratch. The history of the IPCC and IPBES offers both inspiration and cautionary lessons.^32,34^ The IPCC’s credibility derives from meticulous procedures and long assessment cycles.^14^ However, such extended timelines, which are fully appropriate for climate assessments, may be ill-suited to rapidly evolving chemical risks, where regulatory windows can close quickly. The ISP-CWP may therefore need to institutionalize shorter and more flexible assessment cycles without compromising methodological rigor—a balance that earlier panels were not structurally required to strike.^15,35^ Mechanisms such as early notification procedures, regularly updated expert rosters involved, and flexible contribution formats could help strengthen the Panel’s responsiveness while maintaining robust scientific input.^26^ IPBES has demonstrated the value of integrating Indigenous and local knowledge, yet it has also faced challenges related to procedural complexity and uneven participation across regions.^36,37^ Approaches such as dedicated knowledge-holder dialogues, engagement frameworks, inclusive expert nomination procedures, and tailored capacity-building initiatives could help facilitate meaningful participation while maintaining manageable institutional processes.^38^

Communication is another area where the new Panel could further innovate. Expanding communication formats may enhance usability, but risks of fragmenting authority if outputs vary in perceived robustness. Establishing clear hierarchies of deliverables will be essential to preserve epistemic clarity. The IPCC’s Summaries for policymakers remain iconic, but they are also dense documents.^35^ The ISP-CWP, with its mandate to be solutions-focused, has a formal set of deliverables outlined in the draft decision text. These include assessments at different scales, synthesis reports, summaries for policymakers, horizon scans, conceptual frameworks, guidelines, and a variety of communication materials (CRP.5, Annex III, para B, (2–6)).^28^ This portfolio ensures credibility and comparability across science-policy bodies while signaling that the Panel’s impact will depend on more than producing comprehensive reports. To maximize usability, the ISP-CWP could pioneer accessible formats such as interactive dashboards, scenario roadmaps, or digital visualization tools, complementing formal assessments and making scientific insights actionable for policymakers, practitioners, and communities. If the Panel successfully combines the credibility of established processes with the agility of innovative communication, it may fill a long-standing gap.

## From Institutional Design to Governance Practice

The ISP-CWP closes a long-recognized gap in the global environmental governance architecture. Yet closing a gap does not automatically resolve the structural challenges that have historically constrained collective action on chemicals and waste. It draws on procedural lessons from established panels while adapting to the specific context of chemicals and waste governance characterized by fragmented treaty regimes, uneven regulatory capacities across countries, and the presence of strong industrial interests. The Panel’s effectiveness will depend on whether its institutional design translates into sustained authority in practice.

As this Perspective has argued, the Panel’s success will ultimately depend on how effectively it manages a set of persistent institutional tensions—including sustainable financing, safeguarding scientific independence, balancing timeliness with methodological rigor, and ensuring meaningful, inclusive participation across regions. If successful, the ISP-CWP could become a model for other neglected domains where risks are diffuse, data are uneven, and economic stakes are high, such as health, marine ecosystem protection, pandemics and antimicrobial resistance.^39^ If not, the Panel risks becoming an advisory body whose outputs remain marginal to decision-making processes. The coming years will therefore determine whether the ISP-CWP consolidates scientific authority in a fragmented field or remains institutionally peripheral. In this sense, the Panel represents not only a new institution, but a live test of how global governance can organize credible, policy-relevant science in politically sensitive and rapidly evolving areas of environmental risk.

## Acronyms

COI: conflict-of-interest
CRP: conference room paper
IEC: Interdisciplinary Expert Committee
IPBES: Intergovernmental Science-Policy Platform on Biodiversity and Ecosystem Services
IPCC: Intergovernmental Panel on Climate Change
ISP-CWP: Intergovernmental Science-Policy Panel on Chemicals, Waste and Pollution
LMICs: low- and middle-income countries
MEAs: multilateral environmental agreements
OEWG: open-ended working group
UN: United Nations
UNEA: United Nations Environment Assembly
UNEP: United Nations Environment Programme
UNFCCC: United Nations Framework Convention on Climate Change
